# Supplementary figures and images for: Clove Essential Oil and Its Main Constituent, Eugenol, as Potential Natural Antifungals against Candida spp. Alone or in Combination with Other Antimycotics Due to Synergistic Interactions
Source: Molecules. 2022 Dec 26;28(1):215. doi: 10.3390/molecules28010215 (PMC9821947; doi:10.3390/molecules28010215)

## SUPPLEMENTARY FILE

a)

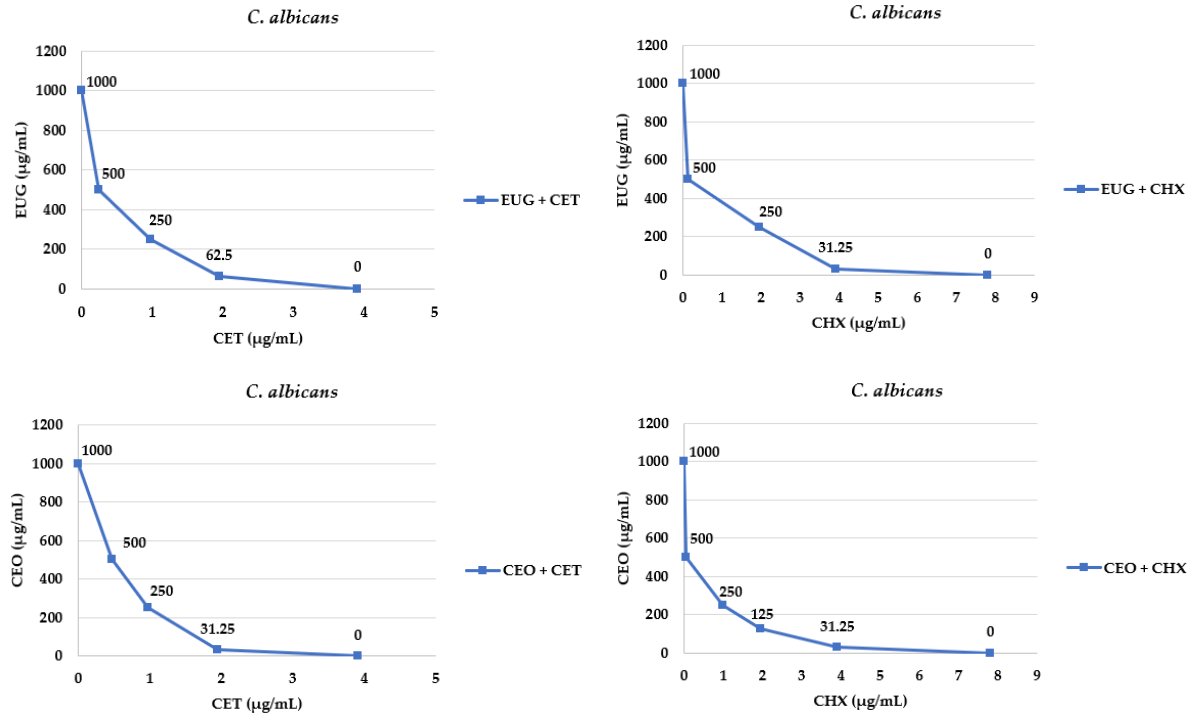

b)

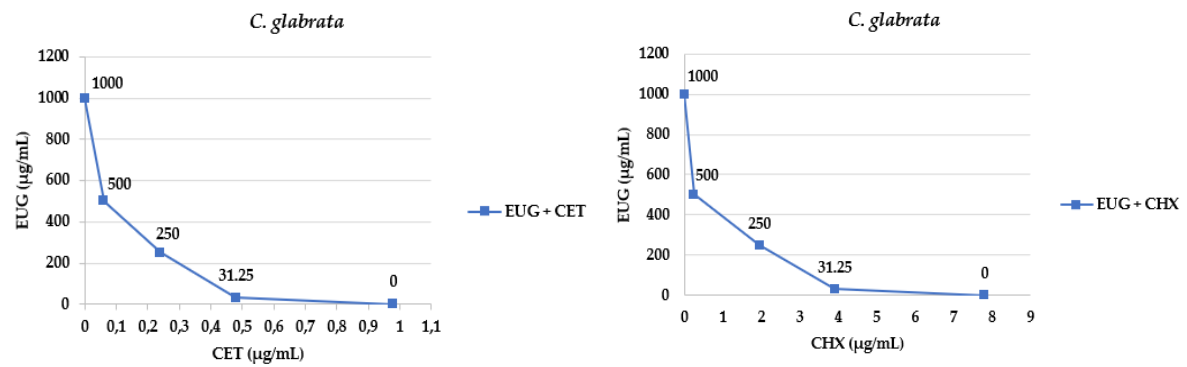

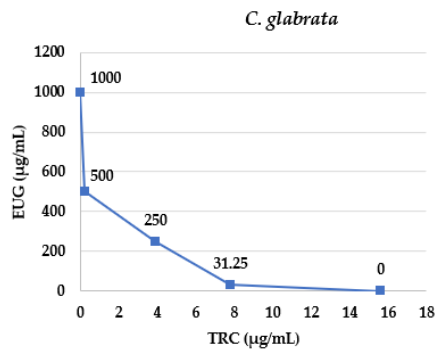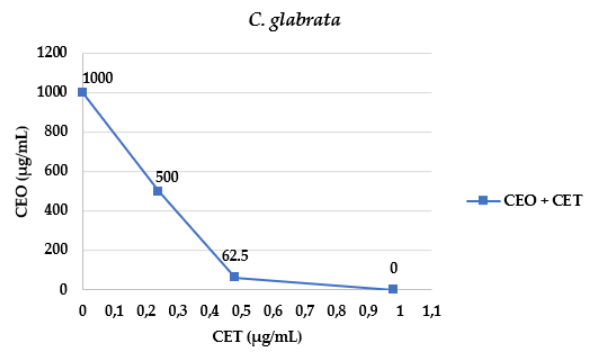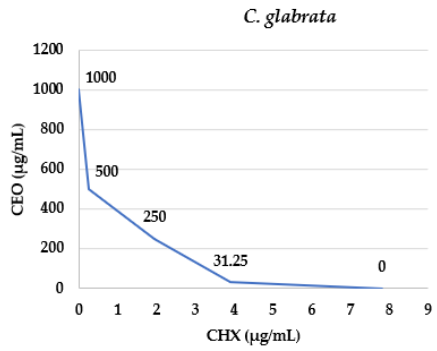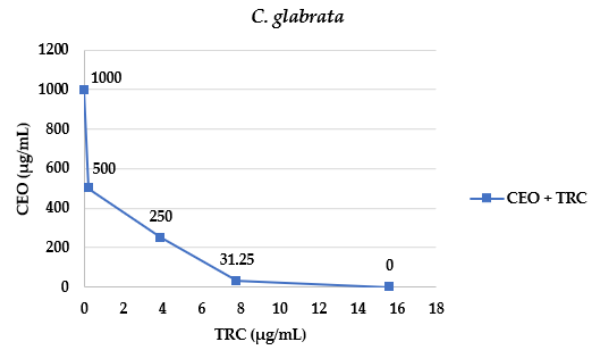

c)

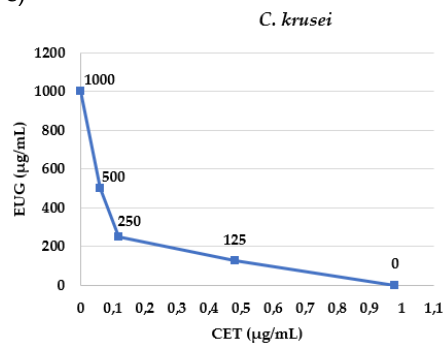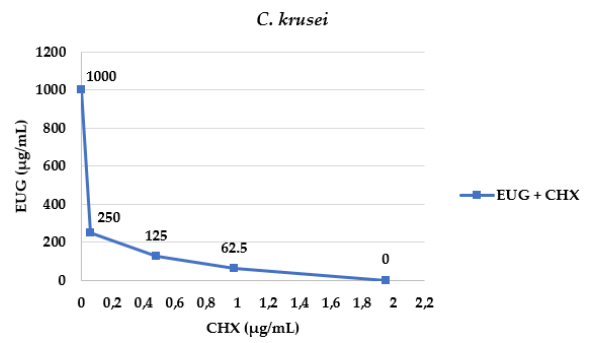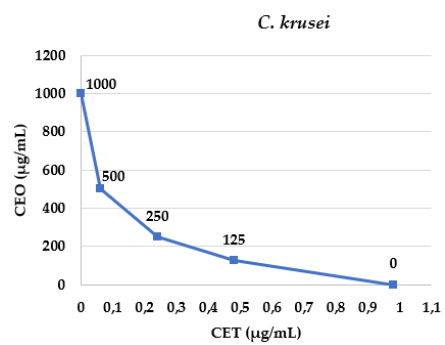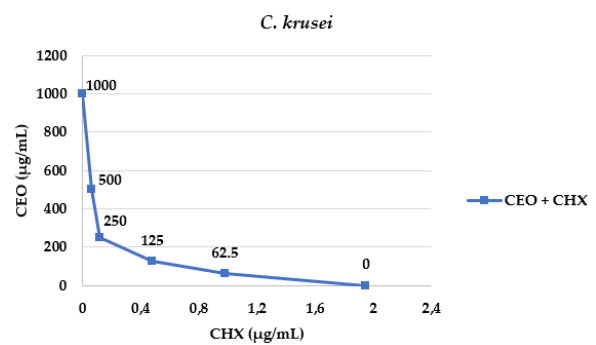

d)

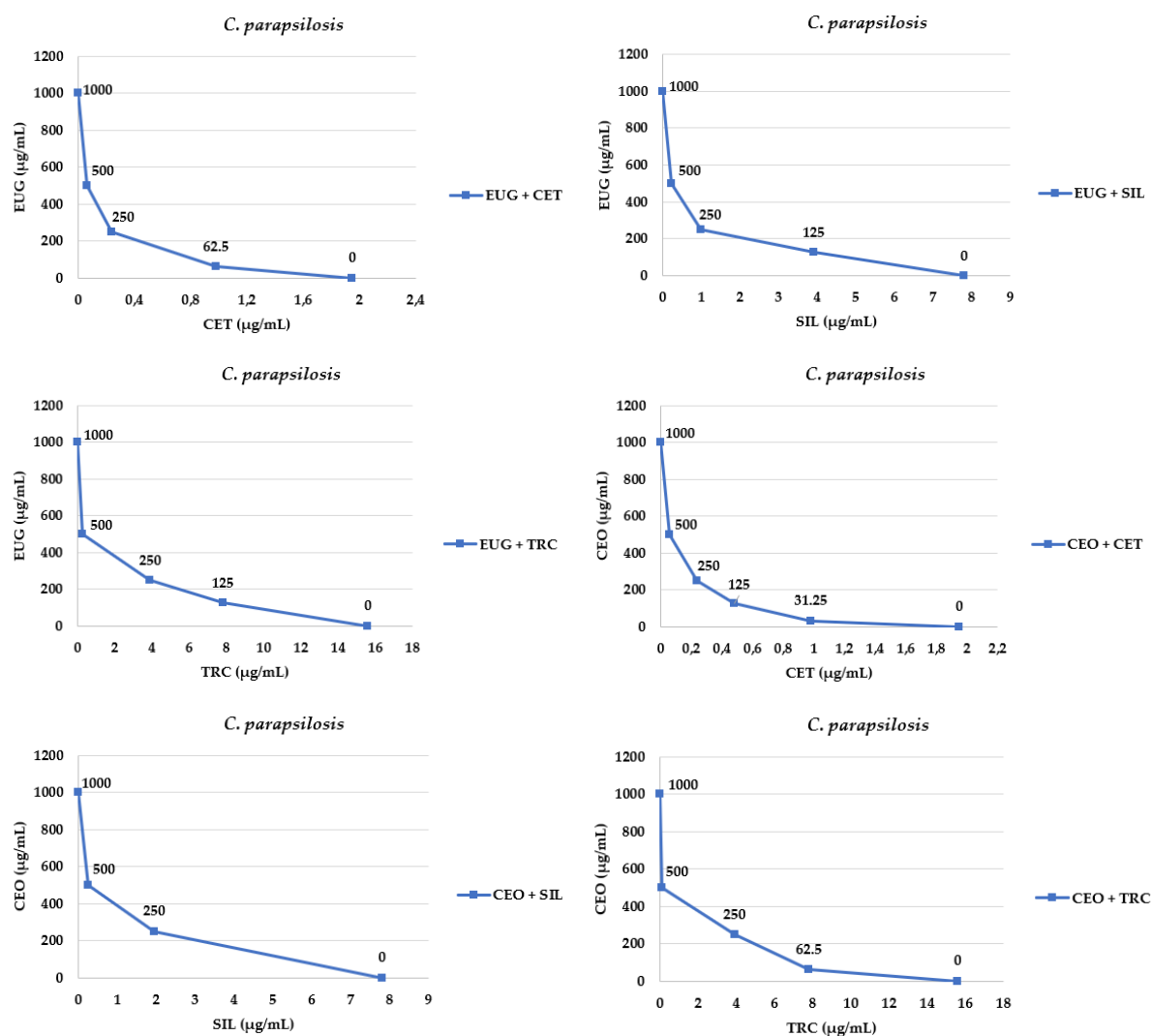

Supplement: Supplementary file 1 [file molecules-28-00215-s001.zip › molecules-2102449-supplementary.pdf]
